# LightAWNet: Lightweight adaptive weighting network based on dynamic convolutions for medical image segmentation

**DOI:** 10.1002/acm2.14584

**Published:** 2024-12-01

**Authors:** Xiaoyan Wang, Jianhao Yu, Bangze Zhang, Xiaojie Huang, Xiaoting Shen, Ming Xia

**Affiliations:** ^1^ School of Computer Science and Technology Zhejiang University of Technology Hangzhou Zhejiang China; ^2^ The Second Affiliated Hospital, School of Medicine Zhejiang University Hangzhou China; ^3^ Stomatology Hospital, School of Medicine Zhejiang University Hangzhou China

**Keywords:** dynamic convolution, lightweight models, medical image segmentation

## Abstract

**Purpose:**

The complexity of convolutional neural networks (CNNs) can lead to improved segmentation accuracy in medical image analysis but also results in increased network complexity and training challenges, especially under resource limitations. Conversely, lightweight models offer efficiency but often sacrifice accuracy. This paper addresses the challenge of balancing efficiency and accuracy by proposing LightAWNet, a lightweight adaptive weighting neural network for medical image segmentation.

**Methods:**

We designed LightAWNet with an efficient inverted bottleneck encoder block optimized by spatial attention. A two‐branch strategy is employed to separately extract detailed and spatial features for fusion, enhancing the reusability of model feature maps. Additionally, a lightweight optimized up‐sampling operation replaces traditional transposed convolution, and channel attention is utilized in the decoder to produce more accurate outputs efficiently.

**Results:**

Experimental results on the LiTS2017, MM‐WHS, ISIC2018, and Kvasir‐SEG datasets demonstrate that LightAWNet achieves state‐of‐the‐art performance with only 2.83 million parameters. Our model significantly outperforms existing methods in terms of segmentation accuracy, highlighting its effectiveness in maintaining high performance with reduced complexity.

**Conclusions:**

LightAWNet successfully balances efficiency and accuracy in medical image segmentation. The innovative use of spatial attention, dual‐branch feature extraction, and optimized up‐sampling operations contribute to its superior performance. These findings offer valuable insights for the development of resource‐efficient yet highly accurate segmentation models in medical imaging. The code will be made available at https://github.com/zjmiaprojects/lightawnet upon acceptance for publication.

## 1 INTRODUCTION

Medical image segmentation plays a crucial role in the medical field by aiding physicians in making accurate and timely diagnoses in complex cases, thereby guiding treatment planning and promoting research. This process involves precisely delineating specific structures or regions within medical images, such as organs, lesions, and tumors, providing essential information for effective medical diagnosis and treatment.^1^, ^2^ Convolutional neural networks (CNNs) and Transformers have emerged as dominant methodologies in the field of medical image analysis.^3^, ^4^ These technologies significantly enhance the accuracy and efficiency of image‐based diagnosis by extracting complex multi‐level features and integrating them effectively. For instance, U‐Net,^5^ proposed and widely used for medical image segmentation tasks, has been the basis for numerous extensions^6^, ^7^, ^8^ and improvements aimed at achieving state‐of‐the‐art performance. 3D CNN,^9^, ^10^ introduced for handling 3D data, performs convolution operations across three dimensions, better capturing spatial features within the data and improving segmentation accuracy. Moreover, the vision transformer (ViT),^11^ leveraging self‐attention mechanisms to capture global image features, has outperformed traditional CNN methods. Despite the remarkable advancements in segmentation performance achieved by these models, they come at the cost of increased network complexity, including a significant rise in the number of parameters (Params) and computational load (GFLOPs).

In order to enhance the efficiency of feature extraction, researchers have been exploring the application of lightweight algorithms within neural networks. These techniques encompass methods such as network pruning,^12^ weight quantization,^13^ and low‐rank approximation.^14^ Common lightweight methods, such as the MobileNet series^15^, ^16^, ^17^ utilize depth‐wise separable convolutions to reduce parameters and computation. Shufflenet^18^ and Shufflenet V2^19^ introduce channel shuffling and channel splitting to enhance and balance the relationships between channels. Ghostnet^20^ and CEModule^21^ employ depth‐wise separable convolutions and a series of simple linear operations to replace some convolution operations, and further reduce FLOPs through group convolutions. BiseNet^22^ and BiseNet V2^23^ acquire spatial and semantic information through two branches, with the latter optimizing the structure and introducing enhanced training strategies. Lightweight Transformer methods have also gained considerable attention, such as Mobile‐Former,^24^ ToPFormer,^25^ and MobileViT,^26^ which combine the strengths of MobileNet and Transformer and demonstrate excellent accuracy. UNeXt^27^ reduces the number of convolution layers and replaces them with mobile tokenization MLPs(multi‐layer perceptrons) to reduce parameters. There are also lightweight neural network methods that leverage contextual information, such as CelNet,^28^ which utilizes depth‐wise separable convolutions and a Siamese network structure to conserve computational resources. Currently, in the latest research on lightweight networks, Lang et al.^29^ proposed a novel approach for capturing global contextual information while preserving spatial details. This method utilizes self‐attention mechanisms to process features and perform fusion reconstruction in the decoder. Liu et al.^30^ proposed a detail enhancement and denoising block (DED) to enhance the accuracy of segmentation for small pathological lesions.

While these lightweight methods reduce the number of parameters and computational complexity to some extent, they generally suffer from the following drawbacks: (1) Limited feature representation capability: Many methods reduce convolution operations and the number of layers, leading to decreased feature representation ability, particularly in handling complex scenes and fine details. (2) Fixed convolution kernel parameters: Traditional convolutional kernels have fixed parameters, making it difficult to adapt to varying input datasets, resulting in suboptimal performance on diverse data. Therefore, our research aims to achieve a balance between maintaining high accuracy and model lightweightness.

Dynamic convolution^31^, ^32^ can adaptively adjust the convolutional kernel weights based on the characteristics of the input data, thereby enhancing the model's ability to capture different features while maintaining low computational complexity. Compared to fixed‐weight convolution operations, dynamic convolution can flexibly adjust the kernel parameters to better suit various input data, improving the model's generalization capability. Therefore, we incorporate dynamic convolution into our method.

Furthermore, to enhance the lightweight model's capability in handling complex image content or fine details, we introduce an efficient spatial attention mechanism and a dual‐branch strategy. The spatial attention mechanism^33^ focuses on important spatial locations in the feature map, effectively capturing key regions in the image and improving the model's ability to recognize detailed features. The dual‐branch strategy^34^ extracts semantic and spatial features separately and then fuses them, significantly improving feature reuse and model performance. Based on these advantages, we designed an efficient inverted bottleneck encoder block that enhances feature extraction precision through the spatial attention mechanism and adopts the dual‐branch strategy for semantic and spatial feature fusion, significantly improving the model's feature reuse capability.

Transposed convolution is commonly used for upsampling in decoder blocks, but traditional transposed convolution often leads to high parameter counts and computational complexity.^35^ To effectively reduce parameter counts and computational complexity, we designed an optimized upsampling operation to replace transposed convolution. Additionally, we introduced a channel attention mechanism^36^ in the decoder blocks to further enrich the feature maps.

In summary, this paper proposes a lightweight neural network based on dynamic convolutions, designed to effectively address the complexity and accuracy challenges in multi‐slice medical image segmentation. The main contributions are as follows:

1. We propose a novel lightweight adaptive weighting network for medical image segmentation based on dynamic convolution, which enhances the feature representation of the model while reducing the model's computational complexity.
2. We design an efficient inverted bottleneck encoder block optimized by spatial attention, and employ a two‐branching strategy to respectively extract detail features and spatial features for fusion, thus greatly improving the reusability of model feature maps.
3. We design a lightweight optimized up‐sampling operation to replace transposed convolution and enrich the feature maps by using channel attention in the decoder block, and then more accurate output is obtained efficiently.
4. Experiments on public datasets demonstrate the effectiveness and efficiency of our proposed method. It achieves state‐of‐the‐art performance on WHS, ISIC, and Kvasir‐SEG datasets requiring only 2.83 million parameters.

## 2 APPROACH

### 2.1 Overview

In this work, we decompose 3D medical images into multiple slices for segmentation tasks. Let a 3D medical image be denoted by $X\in R^{D\times H\times W}$, where D is the number of slices, H and W represent the length and width of each slice respectively. Due to the utilization of dynamic convolutions, the weights of different convolutional kernel parameters are adaptively generated based on the input during model training. Moreover, spatial attention and channel attention are applied to the feature maps in the encoder part (DyC‐IS Block) and decoder part (PFF‐DyC Block), respectively. Since the utilization of dynamic convolutions, depthwise separable convolutions, optimized upsampling operations, and an inverted bottleneck structure, the parameter count and computational complexity are reduced in the Lightweight Adaptive Weighted Network while maintaining high accuracy in segmentation results.

**FIGURE 1:**
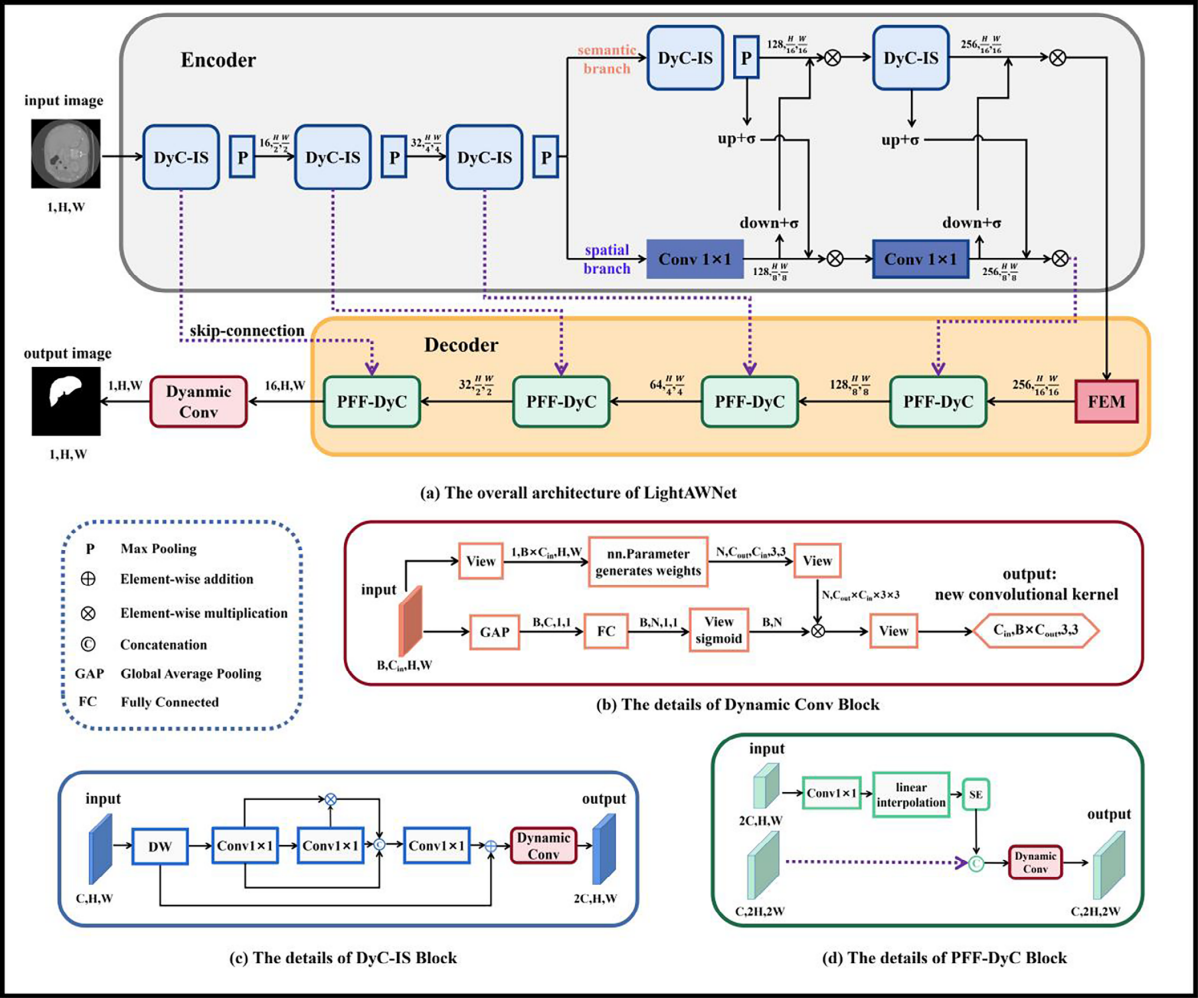
In (a), we show the overall architecture of the proposed LightAWNet, where the FEM is a standard feature enhancement module. In (b) we show the schematic diagram of the dynamic convolution block. Dynamic convolution is applied in each layer of the DyC‐IS block and PFF‐DyC block. (c) and (d) show the detailed structures of these two blocks, respectively.

As shown in Figure 1a, our framework is based on an encoder–decoder structure. In the encoding part, three consecutive DyC‐IS Blocks are employed for feature extraction, followed by max pooling for downsampling after each DyC‐IS Blocks. Subsequently, we divide the network into two branches: the semantic branch and the spatial branch. The semantic branch continues downsampling to extract deeper and more detailed semantic information, while the spatial branch uses convolution to maintain the size of the feature maps while altering the channels, thereby preserving the spatial information of the feature maps. Sigmoid and element‐wise multiplication are employed to update the feature maps and fuse the feature information from both branches. In the decoding part, each PFF‐DyC Block employs lightweight upsampling operations instead of transpose convolution. The features extracted from the encoder stages are input to the PFF‐DyC Block through skip connections for feature fusion. The squeeze and excitation (SE) mechanism is also incorporated to further enhance the features. Finally, the dynamic convolution block is used to output the final results.

### 2.2 Dynamic convolution

Dynamic convolution can dynamically generate convolutional kernel weights, enabling the fitting of more data. Compared to regular convolution, dynamic convolution adds two computational steps: attention weight calculation and dynamic weight fusion. These additional steps are computationally negligible, especially compared to the extensive calculations in regular convolution operations. The working process of dynamic convolution is illustrated in Figure 1b. The input feature map has dimensions $X\in R^{B\times C_{\mathrm{in}}\times H\times W}$, where *B* represents the batch size, *C*_in_ represents the number of input channels, and *H* and *W* represent the height and width of the feature map, respectively.

The dynamic convolution process is divided into two branches. Weight generation branch one: This branch uses global average pooling to convert the feature map dimensions to $B\times C_{\mathrm{in}}\times 1\times 1$. Next, a fully connected layer changes the number of channels to *N*, where *N* represents the number of weights. Using the ‘view’ function in Python libraries, the shape is adjusted to a tensor of B×N. This tensor is processed through a Sigmoid function to generate N weights. Weight Generation Branch Two: This branch primarily generates convolution kernel weights through the `nn.Parameter` function, which initializes the weights. These weights can be learned adaptively by the model during training. Finally, the weights generated by the first branch are linearly combined with the convolution kernel parameters from the second branch to produce new convolution kernel parameters. For each input in the network, the batch size *N* is set to 1. Therefore, the new convolution kernel differs from regular convolution only in its parameters, while the input and output channels and the convolution kernel size remain the same.

Through this method, dynamic convolution can dynamically adjust its parameters based on the characteristics of the input data, enhancing the network's flexibility and adaptability to data while maintaining low computational cost. The adaptability of dynamic convolutions significantly enhances segmentation performance by capturing diverse image features more effectively, thus improving feature representation accuracy. The adaptive weights enable the model to adjust convolution kernels dynamically, boosting robustness and generalization across various input data types and styles. This flexibility also reduces overfitting risk, leading to better test performance. Despite the extra computation for weight generation, dynamic convolutions remain advantageous in terms of parameter count and computational load compared to traditional convolutions. Consequently, adaptive weights allow the model to achieve higher performance with fewer parameters, balancing computational efficiency and segmentation accuracy.

### 2.3 Dual‐branch fusion

This section introduces a weight‐update‐based dual‐branch fusion method aimed at optimizing the integration of feature maps obtained from two different branches, as illustrated in Figure 1a. This method leverages the complementarity between deep and shallow features to enhance the model's ability to comprehensively process semantic and spatial information.

The fusion process is detailed as follows: After three rounds of DyC‐IS block processing and pooling operations, the resulting feature map size is 1/8 of the original image, ensuring that the feature maps have a uniform scale before fusion. In the semantic branch, the feature map is upsampled to 1/8 of the original size using linear interpolation to match the dimensions of the spatial branch's feature map. A Sigmoid function is then applied to generate weights that match the channel number and dimensions of the spatial branch. These weights are used to update the feature map of the spatial branch through element‐wise multiplication, facilitating the flow of information from deep features to shallow features. In the spatial branch, the feature map is downsampled using linear interpolation to match the dimensions of the semantic branch. Similarly, a Sigmoid function is applied to generate weights that match the channel number and dimensions of the semantic branch. These weights are used to update the feature map of the semantic branch through element‐wise multiplication, promoting effective fusion of shallow and deep features.

The advantage of this dual‐branch fusion strategy is that it allows the model to effectively combine features from different processing stages, enhancing the model's understanding and representation of complex image content. By merging shallow and deep features, the semantic and spatial information is better complemented.

### 2.4 DyC‐IS block

The DyC‐IS block is a key innovation in our proposed method, combining depthwise separable convolution and spatial attention mechanisms to enhance the model's ability to capture important features while reducing the number of parameters. The DyC‐IS block employs depthwise separable convolution, a choice for most lightweight neural networks due to its significant reduction in parameter count and computational complexity. In our design, we introduce an “inverted bottleneck” structure inspired by ConvNext,^37^ using fewer channels at the ends of the structure and more channels in the middle. This design not only reduces the number of parameters but also increases the network's non‐linearity, thereby enhancing its expressive capability.

As shown in Figure 1c, the operating procedure is as follows: a 7×7 depthwise separable convolution is applied to the input feature map to reduce the model's computational burden. Although the convolution kernel is larger than the conventional 3×3 kernel, the computational load remains low due to the nature of depthwise separable convolution. Additionally, the 7×7 kernel provides a larger receptive field, aiding in the capture of more comprehensive feature information. Then, a 1×1 convolution is used to expand the number of channels by four times, collecting and combining more feature information, denoted as $X_{1}$. Next, another 1×1 convolution compresses the channels back to one, and a Sigmoid function calculates the weight for each pixel, resulting in $X_{2}$. $X_{1}$ and $X_{2}$ are element‐wise multiplied to update the feature map, and the resulting feature map is concatenated using a Concat operation, forming a richer feature map $X_{3}$. Finally, a 1×1 convolution adjusts the number of channels, and the resulting feature map is element‐wise added to the input feature map of the DyC‐IS block. Thus, the dynamic convolution generates the new feature map.

The operations of the DyC‐IS block can be represented by the following equations:

(1) $$X_{1}=C_{1}\left(\mathrm{DW}\left(X\right)\right),$$

(2) $$X_{2}=X_{1}⊗\sigma \left(C_{2}\left(X_{1}\right)\right),$$

(3) $$X_{3}=C_{3}\left(\mathrm{Cat}\left(X_{1},X_{2}\right)\right),$$

(4) $$Y=\mathrm{DyConv}\left(X⊕X_{3}\right),$$

where the input to the DyC‐IS Block is $X\in R^{C_{in}\times H\times W}$ and the output is $X\in R^{C_{\mathrm{out}}\times H\times W}$, DW represents depthwise convolution, $C_{1}$, $C_{2}$, $C_{3}$ represent three 1×1 convolutions, Cat represents concatenate operation, ⊕ and ⊗ represent element‐wise addition and element‐wise multiplication, DyConv represents dynamic convolution.

### 2.5 PFF‐DyC block

The PFF‐DyC block is an advanced decoding block we propose for upsampling and fusing the low‐resolution features extracted by the encoding part. In the PFF‐DyC block, we forgo the conventional transposed convolution in favor of an improved upsampling technique called “progressive upsampling.” As shown in Figure 1d, in this technique, the processing of the input feature $F_{1}$ involves several steps: First, a 1×1 convolution is applied to halve the number of channels in $F_{1}$, reducing the parameters and computational cost of subsequent operations. Then, linear interpolation is used to upsample the feature map $F_{1}$, achieving the same effect as traditional transposed convolution but with greater efficiency in terms of parameters and computational cost. It is crucial to perform channel compression before linear interpolation to ensure efficiency, as interpolation performed before compression would result in similar parameters and computational cost to those of transposed convolution. After upsampling, a SE module is applied to further enhance the features by recalibrating the inter‐channel dependencies and strengthening the important features. Using skip connections, the output $F_{2}$ from the encoding part is introduced into the PFF‐DyC block and fused with $F_{1}$. The final output is generated through dynamic convolution, resulting in more precise decoding. The specific formulas are as follows:

(5) $$F_{1}^{\prime }=\mathrm{SE}\left(\mathrm{Inter}\left(C\left(F_{1}\right)\right)\right),$$

(6) $$\mathrm{output}=\mathrm{DyConv}\left(\mathrm{Cat}\left(F_{1,}F_{2}\right)\right),$$

where C represents 1×1 convolution, and inter represents linear interpolation operation.

### 2.6 Loss function

Finally, for better effectiveness and applicability, our approach adopts the binary cross‐entropy loss function (BCE) as the training loss, denoted as L. The specific formula is expressed as follows:

(7) $$L=\frac{1}{N}\;\sum_{i=1}^{N}-\left[Y_{i}\log(X_{i}\right)+\left(1-Y_{i}\right)\log\left(1-X_{i}\right)],$$

where L denotes the loss, N denotes the number of slices, i denotes the i‐th slice, $X_{i}$ and $Y_{i}$ denote the segmentation result and ground truth of the i‐th slice, respectively.

## 3 EXPERIMENTS AND RESULTS

### 3.1 Dataset

Our experiments were conducted based on publicly available datasets, including the LiTS2017 dataset^1^, the MM‐WHS dataset subset^2^, the ISIC2018 dataset^3^ and the Kvasir‐SEG dataset.^4^

The LiTS dataset is a liver tumor segmentation challenge dataset jointly released by ISBI 2017 and MICCAI 2017. It consists of 131 abdominal CT scan images, where each CT image and its pixel‐level annotations are stored in the NIfTI format. The resolution of each CT image is 512×512. The dataset was initially divided into training, validation, and testing sets in a ratio of 104:13:14. From each CT image, a portion of two‐dimensional slices and their corresponding labels were extracted, resulting in a final ratio of 10 572:1300:1, 496 for the training, validation, and testing sets, respectively.

The MM‐WHS dataset is a whole‐heart segmentation challenge released by Medical Image Analysis. The data used in the experiment is a subset publicly available from MM‐WHS, consisting of 20 MR images. Due to the limited data, a 4:1 ratio was used to split the 20 MR images into training and testing sets for five‐fold cross‐validation experiments. The images are stored in the NIfTI format with a resolution of 223×191. For convenience in conducting consecutive experiments, the resolution was adjusted to 224 × 224. Similarly, two‐dimensional slices and their corresponding labels were extracted from each three‐dimensional MR image, resulting in a total of 3040 slices for the experiment.

The ISIC dataset is a challenge organized by the International Skin Imaging Collaboration (ISIC), which includes camera‐acquired skin images and corresponding labels for skin lesions. The ISIC 2018 dataset consists of 2594 photographs with varying resolutions. To ensure consistency, all images were resized to 224×224. The dataset was divided into training and testing sets in a 4:1 ratio, and five‐fold cross‐validation experiments were conducted.

The Kvasir‐SEG dataset contains 1000 images of polyps along with their corresponding labels. The images have varying resolutions ranging from 332×487 to 1920×1072 pixels. The image files are encoded in JPEG compression format. In this experiment, all images were uniformly resized to 512×512. The training and testing sets were divided in a 1:1 ratio.

For all datasets, the data in different formats were converted to the npy format, and data normalization was performed, scaling the pixel values between 0 and 1.

### 3.2 Implementation details

The experiments were conducted using the open‐source Python machine learning framework PyTorch on a device equipped with NVIDIA GeForce RTX 2080Ti. To compare the raw performance of each model, no data augmentation was applied.

For the LiTS, WHS, and ISIC datasets, the network training utilized the cross‐entropy loss function. The training utilized the Adaptive Moment Estimation (ADAM) optimization strategy with a learning rate set to 0.0001. Each experiment consisted of 100 iterations, with a validation step performed every 5 epochs. Model weights were saved every 10 epochs for testing purposes.

For the Kvasir‐SEG dataset, due to the small data size and the risk of overfitting, a multi‐cycle cosine annealing learning rate decay strategy was employed. This strategy gradually reduces the learning rate within each cycle using a cosine function, preventing the model from getting stuck in local optima and aiding in finding the global optimum. Additionally, the multi‐cycle learning rate decay promotes stable convergence during the training process. The initial learning rate was set to $10^{-3}$, and the minimum learning rate was $10^{-6}$. The training was conducted for 10 cycles, with each cycle consisting of 20 epochs. The learning rate decreased from the maximum value to the minimum value and then returned to the maximum value within each cycle. In total, 200 epochs were trained.

### 3.3 Evaluation metric

In this paper, we selected various metrics to evaluate the performance of the algorithms. To test the segmentation capability, we used dice similarity coefficient (DSC), false negative rate (FNR), intersection over union (IoU), and under‐segmentation rate (UR) as evaluation metrics.

(8) $$\mathrm{DSC}=\frac{2\mathrm{TP}}{2\mathrm{TP}+\mathrm{FP}+\mathrm{FN}},$$

(9) $$\mathrm{IOU}=\frac{\mathrm{TP}}{\mathrm{TP}+\mathrm{FP}+\mathrm{FN}},$$

(10) $$\mathrm{UR}=\frac{\mathrm{FN}}{\mathrm{TP}+\mathrm{FP}+\mathrm{FN}},$$

(11) $$\mathrm{FNR}=\frac{\mathrm{FN}}{\mathrm{TP}+\mathrm{FN}},$$

where TP (TN) refers to the number of pixels correctly predicted and labeled as positive (negative). Conversely, FP (FN) refers to the number of pixels incorrectly predicted and labeled as positive (negative).

### 3.4 Result

Under the same conditions of GPU, optimizer, and learning rate, we compared our model with existing methods on the LiTS dataset, MM‐WHS dataset, ISIC dataset, and Kvasir‐SEG dataset. Given that our model adopts an encoder–decoder structure, we compared it with similar architectures such as U‐Net, CE‐Net,^38^ and SiamUNet. Additionally, we included comparisons with classic lightweight methods like GhostNet, ShuffleNet, the MobileNet series, BiseNet V2, and the latest lightweight models Mobile‐Former and UNeXt. To demonstrate the robustness of our proposed model, we also considered the popular Transformer‐based network ViT as a comparison.

This section is divided into three parts: the first part focuses on the lightweight characteristics of the model, the second part addresses its performance, and the third part provides visualizations.

#### 3.4.1 Lightweight characteristics

In this study, we focused on the lightweight design of the model to reduce hardware requirements in practical applications. We compared our model with existing lightweight network models such as GhostNet, ShuffleNet, and the MobileNet series, as well as traditional larger models like U‐Net and SiamU‐Net. The results indicate that our LightAWNet has significant advantages in terms of parameter count and computational cost. As shown in Table 1, on the LiTS dataset, LightAWNet requires only 2.83 M parameters and 5.64 GFLOPs. Compared to U‐Net, LightAWNet not only outperforms U‐Net in segmentation performance but also reduces Params and FLOPs by approximately 28.21 M and 178.68G, respectively.

**TABLE 1:** Comparison results of segmentation performance on the LiTS dataset.

Method	DSC↑	FNR↓	IoU↑	UR↓	Params↓	GFLOPs↓	Time↓
GhostNet	0.8273	0.1836	0.7659	0.1755	5.98 M	3.64	0.712 s
ShuffleNet V2	0.8316	0.1582	0.7762	0.1441	15.69 M	9.99	1.026 s
MobileNet	0.8518	0.1307	0.8003	0.1113	15.11 M	166.92	2.134 s
MobileNet V2	0.8645	0.1146	0.8132	0.1046	4.68 M	3.38	0.773 s
MobileNet V3	0.8385	0.1063	0.776	0.0938	1.45 M	1.25	0.372 s
Mobile‐Former	0.8668	0.1255	0.7975	0.1061	10.86 M	6.5	1.643 s
UNeXt	0.8624	0.0857	0.7894	0.0813	**0.25 M**	**0.39**	**0.283 s**
BiSeNet V2	0.7481	0.2546	0.6860	0.2368	5.47 M	10.24	1.322 s
ViT	0.8733	0.1375	0.8077	0.1196	85.41 M	106.8	4.71 s
U‐Net	0.8913	0.1208	0.8480	0.1174	31.04 M	184.32	1.92 s
CE‐Net	0.8745	0.0912	0.8067	0.0718	29.0 M	23.5	1.617 s
SiamU‐Net	0.9221	0.0844	0.8837	0.0814	49.92 M	203.62	4.531 s
CeLNet	**0.9233**	**0.0781**	**0.8855**	**0.0715**	5.18 M	16.28	1.114 s
LightAWNet	0.9141	0.0818	0.8675	0.0746	2.83 M	5.64	0.575 s

*Note*: Best results are in bold; our results are in blue. An upward arrow indicates higher values represent better performance, while a downward arrow indicates the opposite.

Abbreviations: DSC, dice similarity coefficient; FNR, false negative rate; IoU, intersection over union; UR, under‐segmentation rate; ViT, vision transformer.

Furthermore, on the WHS and ISIC datasets, as shown in Table 4, LightAWNet has 2.83 M parameters and 1.03 GFLOPs. According to Tables 2 and 3, LightAWNet surpasses other methods in segmentation metrics. In the comparative experiment on the Kvasir‐SEG dataset, shown in Table 5, ViT and Mobile‐Former were excluded due to severe overfitting issues caused by the small sample size. The results demonstrate that LightAWNet still performs exceptionally well on small sample datasets, requiring only 2.83 M parameters and 5.64 GFLOPs, but significantly outperforming both lightweight and traditional methods in segmentation metrics.

**TABLE 2:** Comparison results of segmentation performance on the WHS dataset.

Method	DSC↑	FNR↓	IoU↑	UR↓
GhostNet	0.7193 ± 0.0759	0.2648 ± 0.1494	0.6075 ± 0.0850	0.2953 ± 0.1202
ShuffleNet V2	0.7483 ± 0.0708	0.2700 ± 0.1133	0.6423 ± 0.0776	0.2412 ± 0.1145
MobileNet	0.7533 ± 0.0499	0.2525 ± 0.0813	0.6481 ± 0.0571	0.2216 ± 0.0837
MobileNet V2	0.7269 ± 0.0454	0.2614 ± 0.0863	0.6179 ± 0.0490	0.2238 ± 0.0849
MobileNet V3	0.7256 ± 0.0443	0.2190 ± 0.0968	0.6175 ± 0.0483	0.1788 ± 0.0916
Mobile‐former	0.7615 ± 0.0700	0.2341 ± 0.1220	0.6621 ± 0.0786	0.2076 ± 0.1213
UNeXt	0.7526 ± 0.0324	0.2230 ± 0.0688	0.6459 ± 0.0403	0.1899 ± 0.0646
BiSeNet V2	0.7495 ± 0.547	0.2646 ± 0.0821	0.6537 ± 0.0564	0.2391 ± 0.0839
ViT	0.7437 ± 0.0345	0.2199 ± 0.0793	0.6197 ± 0.0456	0.1887 ± 0.0812
U‐Net	0.7788 ± 0.0437	0.2363 ± 0.0840	0.6807 ± 0.0495	0.2130 ± 0.0871
CE‐Net	0.7727 ± 0.0680	0.2362 ± 0.1050	0.6723 ± 0.0773	0.2191 ± 0.1242
SiamU‐Net	0.7967 ± 0.0673	0.2404 ± 0.0950	0.7048 ± 0.0735	0.2226 ± 0.0953
CelNet	0.7895 ± 0.0322	0.2180 ± 0.0554	0.6898 ± 0.0389	0.1775 ± 0.0543
LightAWNet	**0.8142 ± 0.0404**	**0.1997 ± 0.0628**	**0.7193 ± 0.0470**	**0.1760 ± 0.0552**

*Note*: Best results are in bold; our results are in blue. An upward arrow indicates higher values represent better performance, while a downward arrow indicates the opposite.

Abbreviations: DSC, dice similarity coefficient; FNR, false negative rate; IoU, intersection over union; UR, under‐segmentation rate; ViT, vision transformer.

**TABLE 3:** Comparison results of segmentation performance on the ISIC dataset.

Method	DSC↑	FNR↓	IoU↑	UR↓
GhostNet	0.7944 ± 0.0613	0.2069 ± 0.0692	0.7024 ± 0.0659	0.1986 ± 0.0669
ShuffleNet V2	0.7829 ± 0.0638	0.2067 ± 0.0516	0.6858 ± 0.0729	0.1956 ± 0.0468
MobileNet	0.7564 ± 0.0901	0.1979 ± 0.0424	0.6555 ± 0.1014	0.1781 ± 0.0321
MobileNet V2	0.7656 ± 0.0594	0.2030 ± 0.0588	0.6610 ± 0.0665	0.1906 ± 0.0551
MobileNet V3	0.7722 ± 0.0673	0.2111 ± 0.0528	0.6725 ± 0.0747	0.1987 ± 0.0488
Mobile‐former	0.8240 ± 0.0455	0.1686 ± 0.0538	0.7377 ± 0.0549	0.1622 ± 0.0527
UNeXt	0.7956 ± 0.0658	0.1770 ± 0.0646	0.6997 ± 0.0758	0.1666 ± 0.0615
BiSeNet V2	0.8251 ± 0.0564	0.1558 ± 0.0532	0.7359 ± 0.0672	0.1480 ± 0.0494
ViT	0.7769 ± 0.0665	0.2105 ± 0.0575	0.6722 ± 0.0762	0.1933 ± 0.0511
U‐Net	0.8347 ± 0.0558	0.1199 ± 0.0190	0.7473 ± 0.0675	0.1117 ± 0.0181
CE‐Net	0.8300 ± 0.0581	0.1510 ± 0.0326	0.7466 ± 0.0739	0.1442 ± 0.0287
SiamU‐Net	0.8392 ± 0.0514	0.1571 ± 0.0478	0.7557 ± 0.0598	0.1506 ± 0.0460
CelNet	0.8401 ± 0.0542	0.1338 ± 0.0358	0.7554 ± 0.0648	0.1287 ± 0.0315
LightAWNet	**0.8662 ± 0.0387**	**0.1127 ± 0.0374**	**0.7897 ± 0.0469**	**0.1061 ± 0.0342**

*Note*: Best results are in bold; our results are in blue. An upward arrow indicates higher values represent better performance, while a downward arrow indicates the opposite.

Abbreviations: DSC, dice similarity coefficient; FNR, false negative rate; IoU, intersection over union; UR, under‐segmentation rate; ViT, vision transformer.

**TABLE 4:** Comparison results of lightweight metrics on the MM‐WHS dataset and ISIC dataset.

Method	Params↓	GFLOPs↓	Time↓	Method	Params↓	GFLOPs↓	Time↓
GhostNet	5.98 M	0.7	0.489 s	BiSeNet V2	5.47 M	1.96	0.445 s
ShuffleNet V2	15.69 M	1.91	0.681 s	ViT	85.41 M	17.45	2.267 s
MobileNet	15.11 M	31.95	0.882 s	U‐Net	31.04 M	35.28	0.867 s
MobileNet V2	4.68 M	0.65	0.484 s	CE‐Net	29.0 M	4.5	0.724 s
MobileNet V3	1.45 M	0.27	0.227 s	SiamU‐Net	49.92 M	38.97	1.578 s
Mobile‐former	10.86 M	1.25	1.042 s	CelNet	5.18 M	3.12	0.458 s
UNeXt	**0.25 M**	**0.08**	**0.134 s**	LightAWNet	2.83 M	1.03	0.282 s

*Note*: As both datasets have a similar number of slices, and the size of the slices is 224 × 224, the model's parameter count and GFLOPs are identical. The best results are indicated in bold. Our results are displayed in blue font. An upward arrow indicates that a higher value represents better model performance, while a downward arrow indicates the opposite.

Abbreviations: ISIC, International Skin Imaging Collaboration; ViT, vision transformer.

**TABLE 5:** Comparison results of segmentation performance on the Kvasir‐SEG dataset.

Method	DSC↑	FNR↓	IoU↑	UR↓	Params↓	GFLOPs↓	Time↓
GhostNet	0.484	0.4822	0.3426	0.3559	5.98 M	3.64	0.712 s
ShuffleNet V2	0.5422	0.4737	0.4549	0.4079	15.69 M	9.99	1.026 s
MobileNet	0.5681	0.4637	0.4492	0.4183	15.11 M	166.92	2.134 s
MobileNet V2	0.6546	0.3717	0.5485	0.3368	4.68 M	3.38	0.773 s
MobileNet V3	0.6725	0.3374	0.5678	0.2981	1.45 M	1.25	0.372 s
UNeXt	0.618	0.3493	0.4997	0.2842	**0.25 M**	**0.39**	**0.283 s**
BiSeNet V2	0.578	0.4215	0.4724	0.3515	5.47 M	10.24	1.322 s
U‐Net	0.6744	0.3325	0.557	0.2868	31.04 M	184.32	0.867 s
CE‐Net	0.6462	0.363	0.5359	0.3227	29.0 M	23.5	0.724 s
SiamU‐Net	0.6527	0.3571	0.5278	0.3019	49.92 M	203.62	1.578 s
CeLNet	0.642	0.3793	0.5126	0.3118	5.18 M	16.28	0.458 s
LightAWNet	**0.6978**	**0.2554**	**0.5818**	**0.2053**	2.83 M	5.64	0.575 s

*Note*: Best results are in bold; our results are in blue. An upward arrow indicates higher values represent better performance, while a downward arrow indicates the opposite.

Abbreviations: DSC, dice similarity coefficient; FNR, false negative rate; IoU, intersection over union; UR, under‐segmentation rate.

Compared to the latest lightweight models such as Mobile‐Former and UNeXt, LightAWNet maintains low Params and low FLOPs while exhibiting highly competitive segmentation performance. Across all four datasets, LightAWNet's segmentation metrics significantly surpass those of Mobile‐Former and UNeXt.

#### 3.4.2 Performance

As shown in Tables 1, 2, 3, 4, 5, LightAWNet outperforms various classical and state‐of‐the‐art lightweight models on the LiTS, MM‐WHS, ISIC, and Kvasir‐SEG datasets. Specifically, on the LiTS dataset, compared to the classical U‐Net, LightAWNet improves the DSC by 2.28%, reduces the FNR by 3.9%, increases the IoU by 1.95%, and decreases the UR by 4.28%. Additionally, while LightAWNet's segmentation accuracy is comparable to CeLNet and SiamU‐Net, it has a clear advantage in lightweight metrics.

On the MM‐WHS and ISIC datasets, we conducted five‐fold cross‐validation to verify the model's consistency and reliability. The results show that LightAWNet surpasses the second‐best performance values across all segmentation metrics. Specifically, on the MM‐WHS dataset, compared to the second‐best performance, LightAWNet improves the DSC by 1.75%, reduces the FNR by 1.83%, increases the IoU by 1.45%, and decreases the UR by 0.15%. On the ISIC dataset, LightAWNet improves the DSC by 2.61%, reduces the FNR by 2.11%, increases the IoU by 3.4%, and decreases the UR by 0.56%, compared to the second‐best performance.

Furthermore, LightAWNet performs exceptionally well on the small sample Kvasir‐SEG dataset. Compared to the second‐best performance, LightAWNet improves the DSC by 2.53%, reduces the FNR by 7.71%, increases the IoU by 1.4%, and decreases the UR by 7.53%.

#### 3.4.3 Visualization

**FIGURE 2:**
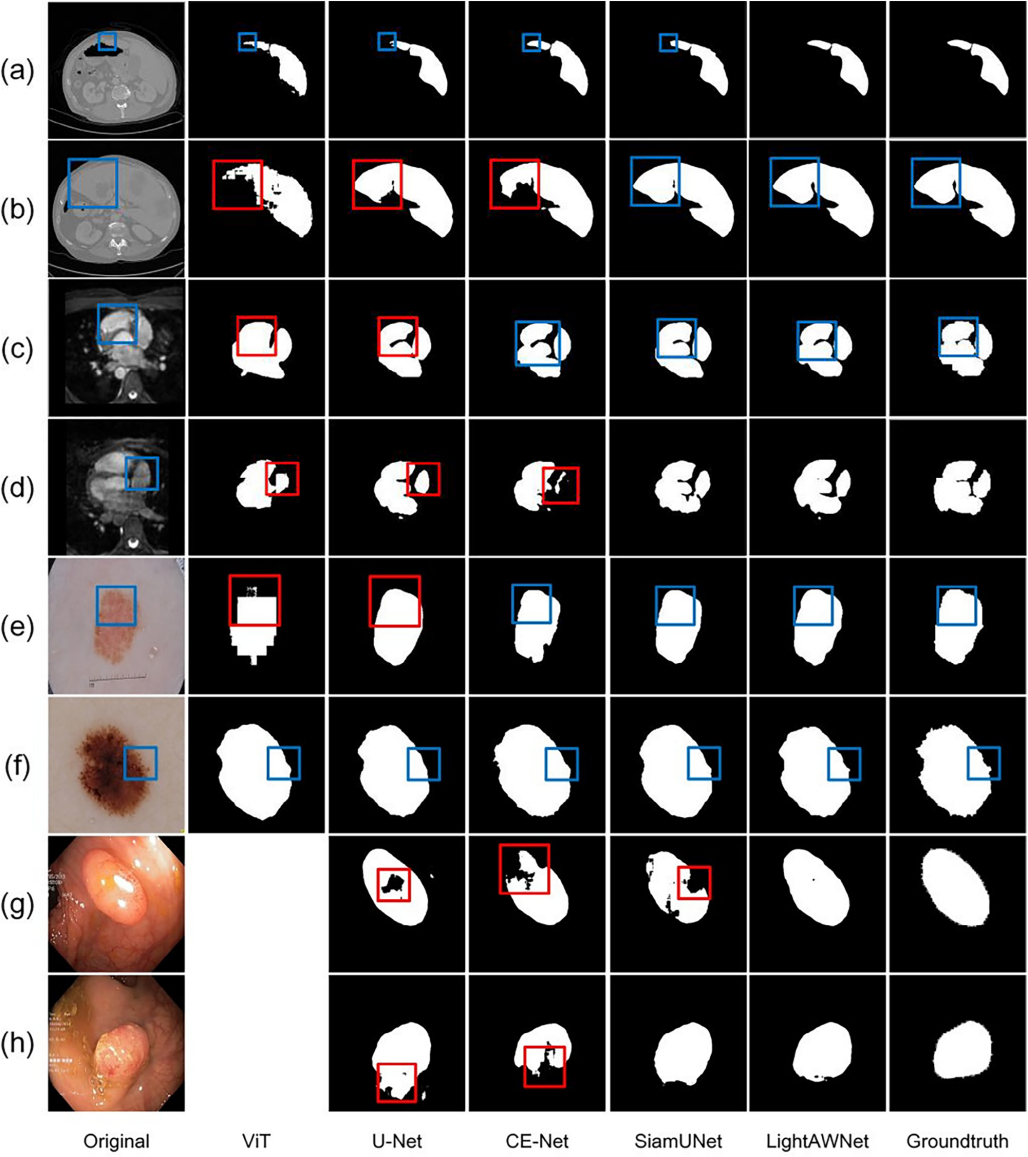
Visual comparisons with common methods. The rows in the figure correspond to the following datasets: Rows (a) and (b) illustrate examples from the LiTS dataset; rows (c) and (d) present examples from the WHS dataset; rows (e) and (f) display examples from the ISIC dataset; and rows (g) and (h) depict examples from the Kvasir‐SEG dataset. Columns indicate the segmentation methods applied: the first column contains the original images, columns two to five show the segmentation results from conventional methods, including ViT, U‐Net, CE‐Net, and SiamU‐Net, respectively. The sixth column presents segmentation outcomes from the proposed LightAWNet model, and the final column provides the ground truth labels. The color‐coded boxes facilitate a comparative analysis: blue boxes highlight detailed segmentation results across different models, while red boxes emphasize notable segmentation errors, aiding in the evaluation of model performance differences. Due to severe overfitting issues, the ViT model could not effectively train on the Kvasir‐SEG dataset, leaving corresponding cells blank in that column. ISIC, International Skin Imaging Collaboration; ViT, vision transformer.

**FIGURE 3:**
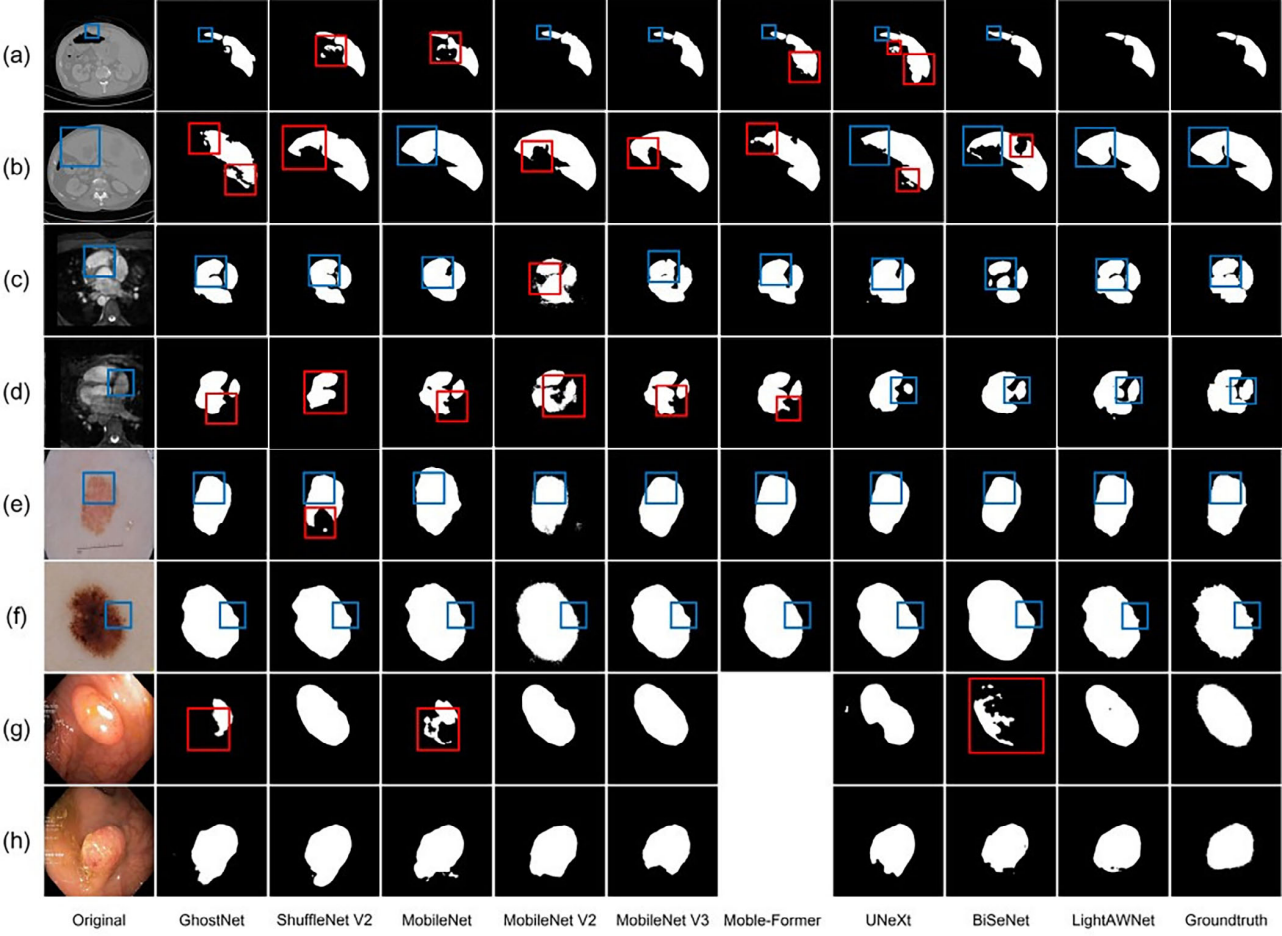
Visual comparisons with lightweight methods. The rows in the figure correspond to the following datasets: Rows (a) and (b) show examples from the LiTS dataset; rows (c) and (d) display examples from the WHS dataset; rows (e) and (f) present examples from the ISIC dataset; and rows (g) and (h) depict examples from the Kvasir‐SEG dataset. The columns represent the segmentation methods: the first column contains the original images; columns two to nine display segmentation results from lightweight methods, including GhostNet, ShuffleNet V2, MobileNet, MobileNet V2, MobileNet V3, Mobile‐Former, UNetX, and BiSeNet. The tenth column presents the segmentation results of the proposed LightAWNet model, while the final column shows the ground truth labels. The color‐coded boxes provide a focused comparative analysis of model performance: blue boxes highlight detailed segmentation outputs across various models, while red boxes mark significant segmentation errors, aiding in the evaluation of model performance differences. Due to severe overfitting, the Mobile‐Former model failed to train effectively on the Kvasir‐SEG dataset, leaving the corresponding cells blank in that column. It can be seen that our method outperforms other models especially in more complex medical images. ISIC, International Skin Imaging Collaboration.

Figures 2 and 3 present a visual comparison of the prediction results of different models, the original images, and the corresponding labels. Figure 2 shows the results of conventional methods such as ViT, U‐Net, CE‐Net, and SiamU‐Net, while Figure 3 displays the results of lightweight methods such as GhostNet, ShuffleNetV2, and the MobileNet series. In both figures, rows represent datasets, and columns represent methods. Rows (a) and (b) show examples from the LiTS dataset, rows (c) and (d) show examples from the WHS dataset, rows (e) and (f) show examples from the ISIC dataset, and rows (g) and (h) show examples from the Kvasir‐SEG dataset. Blue boxes highlight detailed segmentation results of different models, while red boxes emphasize some noticeable segmentation errors. Due to severe overfitting issues, ViT and Mobile‐Former were unable to train on the Kvasir‐SEG dataset, resulting in blank parts in the comparison.

From Figures 2 and 3, it is evident that the proposed LightAWNet performs exceptionally well across multiple datasets. Particularly on the Kvasir‐SEG dataset, other comparative methods exhibit significant errors, whereas LightAWNet shows remarkable improvements across all evaluation metrics. This demonstrates that LightAWNet has strong adaptability to various datasets and performs well on both large and small datasets.

### 3.5 Ablation study

We conducted ablation experiments on the key components, dynamic convolutions, and upsampling methods in the model on the LiTS dataset, as follows:

Since the proposed method is based on dynamic convolutions, we first conducted partial ablation experiments on dynamic convolutions. The generation of dynamic convolutions includes attention weight generation and dynamic weight generation. Table 6 shows the results of the ablation experiment on the number of attention weights required during the generation of dynamic convolutions. Overall, it can be observed that as the number of attention weights increases from less than 4, the performance of the model gradually improves. However, when the number of attention weights exceeds 4, the performance shows oscillating degradation. This is because with too few attention weights, the model may not fully utilize the attention mechanism to focus on important features, resulting in poor performance. On the other hand, when there are too many attention weights, the model may overly focus on training details, such as irrelevant features and noise, introducing noise during training. Moreover, having too many weights can lead to overfitting, resulting in a decrease in segmentation performance.

**TABLE 6:** Comparison of the number of attention weights in dynamic convolution.

The number of attention weights	DSC↑	The number of attention weights	DSC↑
1	0.8603	6	0.8796
2	0.8755	8	0.8890
3	0.8897	10	0.8763
4	**0.9141**	12	0.8721
5	0.8833	15	0.8732

*Note*: The best results are indicated in bold.

Abbreviation: DSC, dice similarity coefficient.

When the number of attention weights is 5, the model has a computational cost of 5.65G operations and 3.43 M parameters. When the number of attention weights is 16, the computational cost is still 5.65G operations, but the number of parameters increases to 9.9 M. The overall computational cost of the model is almost negligible with respect to the number of attention weights. However, as the number of attention weights increases, the parameter count of the model increases significantly. Through continuous adjustment and research on this hyperparameter, we found that using 4 attention weights for dynamic convolutions achieves optimal performance with a relatively small parameter count.

Table 7 presents the ablation experiment on the usage of dynamic convolutions. Conv represents the model in which all dynamic convolutions are replaced with regular convolutions, while Conv 2× represents the model where the number of channels in Conv is doubled. It can be observed that when all dynamic convolutions in the model are replaced with regular convolutions, the model fails to adapt to the dataset and suffers from underfitting, resulting in poor learning. However, when the number of channels is increased, the model can learn properly. Nevertheless, the segmentation performance is noticeably inferior to that of LightAWNet, indicating the excellent fitting capability of dynamic convolutions. Compared to regular convolutions, dynamic convolutions significantly enhance the model's performance without a substantial increase in parameter count and computational cost.

**TABLE 7:** Ablation study of dynamic convolution.

Method	DSC↑	FNR↓	IoU↑	UR↓	Params↓	GFLOPs↓
Conv	0.6594	0.1243	0.5289	0.0998	**1.1 M**	**5.55**
Conv 2×	0.8964	0.1034	0.8355	0.0907	4.26 M	21.15
LightAWNet	**0.9141**	**0.0818**	**0.8675**	**0.0746**	2.83 M	5.64

Abbreviations: DSC, dice similarity coefficient; FNR, false negative rate; IoU, intersection over union; UR, under‐segmentation rate.

Table 8 displays the results of the ablation experiments on the SE module and feature enhancement module (FEM). In this comparative experiment, the base network without adding the FEM and SE module is used as the baseline. It can be observed that by adding the SE module, the parameter count of the model hardly increases, while the computational cost increases by 0.14G operations. By adding the FEM, the parameter count and computational cost of the model increase by 0.07 M and 0.38G operations, respectively. Both modules contribute to performance improvement. Specifically, when the SE module is added, compared to the base model, the DSC increases by 1.53%, FNR decreases by 1.66%, IoU increases by 2.18%, and UR decreases by 1.53%. When the FEM is added, compared to the base model, the DSC increases by 2.02%, FNR decreases by 2.63%, IoU increases by 2.32%, and UR decreases by 2.4%. When both the SE module and FEM are added, compared to the base model, the DSC increases by 2.84%, FNR decreases by 4.26%, IoU increases by 3.89%, and UR decreases by 4.02%. Taking all factors into consideration, the base+SE+FEM configuration is chosen as the final model, namely LightAWNet.

**TABLE 8:** Ablation study of SE and FEM module.

Method	DSC↑	FNR↓	IoU↑	UR↓	Params↓	GFLOPs↓
Base	0.8857	0.1244	0.8286	0.1148	**2.76 M**	**5.12**
Base+SE	0.9010	0.1078	0.8504	0.0995	2.76 M	5.26
Base+FEM	0.9059	0.0981	0.8518	0.0908	2.83 M	5.5
Base+SE+FEM	**0.9141**	**0.0818**	**0.8675**	**0.0746**	2.83 M	5.64

Abbreviations: DSC, dice similarity coefficient; FNR, false negative rate; IoU, intersection over union; SE, squeeze and excitation; UR, under‐segmentation rate.

The ablation experiment results for the progressive upsampling strategy of the PFF‐DyC Block in the decoder section can be referred to in Table 9. “Trans” represents using transpose convolution for upsampling, “size→channel” means processing the feature map size before the channel, and “channel→size” means processing the feature map channel before size. Since the parameter count of convolutional operations is independent of the feature map size, the parameter count difference between “size→channel” and “channel→size” is minimal. However, the computational cost is directly proportional to the feature map size, so the “size→channel” the approach requires significantly more computation compared to “channel→size”. Transpose convolution, while performing upsampling, also alters the channels, and each channel's convolutional kernel needs to be learned. Therefore, the parameter count is larger but the computational cost is smaller compared to the “size→channel” approach. The upsampling approach in this method, achieved by channel compression to aggregate rich features, not only yields high accuracy but is also lightweight.

**TABLE 9:** Ablation study of upsampling mode.

Method	DSC↑	FNR↓	IoU↑	UR↓	Params↓	GFLOPs↓
Trans	0.9042	0.0972	0.8564	0.0853	2.97 M	6.05
Size→channel	0.8840	0.1060	0.8239	0.0863	2.84 M	6.11
Channel→size	**0.9141**	**0.0818**	**0.8675**	**0.0746**	**2.83 M**	**5.64**

Abbreviations: DSC, dice similarity coefficient; FNR, false negative rate; IoU, intersection over union; UR, under‐segmentation rate.

## 4 DISCUSSION

To address the high equipment requirements often necessary for achieving accuracy in traditional segmentation methods, we approached the problem from a lightweight perspective. Conventional convolutional methods use fixed convolution kernels during training, often requiring additional convolution layers or increased model channel capacity to accommodate a broader range of data. However, these adjustments typically lead to a significant increase in model parameters and computational complexity. To mitigate this issue, our study proposes a lightweight medical image segmentation method based on dynamic convolution. By incorporating adaptive convolution kernel generation through dynamic convolution, we introduced multiple dynamic convolution layers in the network, expanding the model's capacity to handle a broader range of data. Simultaneously, we reduced the number of model channels appropriately, significantly lowering the parameters and computational requirements.

During the construction and training of the lightweight medical image segmentation network, LightAWNet, we faced multiple challenges. The primary challenge was to ensure that the model's lightweight nature did not compromise its segmentation performance. Although LightAWNet is designed as a lightweight model, handling larger datasets or high‐resolution images may still require high‐performance computing resources to ensure reasonable training times. The optimization of dynamic convolution layers and hyperparameter tuning significantly impacts the training convergence speed and final performance. For instance, through iterative hyperparameter adjustments, we achieved optimal performance with a dynamic convolution weight count of four while maintaining a relatively small parameter count.

Regarding hardware and software configuration, our experiments were conducted using an NVIDIA GeForce RTX 2080Ti GPU. Researchers aiming to replicate our findings will need similar or higher‐performance GPUs to complete training within a reasonable timeframe. The model was implemented in the PyTorch framework, and normalization, that is, normalizing input image pixel values to the range of 0–1, is crucial for LightAWNet's performance, significantly improving convergence during training. Throughout the training process, we performed multiple iterations and cycles, such as training for 100 epochs on the LiTS, WHS, ISIC, and Kvasir‐SEG datasets, with periodic validation steps. We employed the ADAM optimization strategy and adjusted the learning rate schedule to optimize performance.

In the context of specific clinical scenarios, such as the key task of liver tumor segmentation in medical imaging, there is often a need for precise and efficient segmentation techniques to assist in treatment planning. The primary advantages of our LightAWNet are reflected in its lightweight and efficient design, featuring only 2.83 million parameters, which allows it to operate on low‐end hardware without sacrificing performance. This makes it particularly suitable for deployment in resource‐limited medical environments, such as healthcare institutions with limited access to high‐performance computing resources. Additionally, its dual‐branch feature fusion strategy and spatial attention mechanism enable LightAWNet to achieve high segmentation accuracy, which is essential for the precise localization of liver tumors or lesions, contributing to more accurate diagnosis and treatment planning. However, potential challenges arise when transitioning from controlled datasets like LiTS2017 to real clinical data, as model generalization may be affected by variations in scan quality and patient diversity.

Nevertheless, LightAWNet has certain limitations. For example, in extreme medical cases (such as very rare or abnormal medical image features), the model may not provide optimal segmentation results. Additionally, while performing excellently in 2D medical image segmentation, the model's performance requires improvement when directly handling 3D medical images.

Future research will focus on addressing these limitations. We plan to explore more efficient optimization techniques to reduce training time and improve convergence speed. Furthermore, efforts will be made to enhance LightAWNet's capability in handling 3D medical images, particularly by developing more effective 3D dynamic convolution and feature fusion strategies to improve the model's performance in 3D image segmentation tasks.

## 5 CONCLUSION

In this paper, we have explored the advantages and limitations of traditional segmentation methods and lightweight methods. It proposes a lightweight medical image segmentation network based on dynamic convolutions. By incorporating dynamic convolutions at each layer of the network, the model is better able to adapt to variations in input data, thereby enhancing its adaptability and generalization. Furthermore, spatial attention is applied to feature maps during the encoding phase to enhance the “bottleneck” structure, improving the model's feature extraction capabilities while maintaining a low parameter count. The upsampling strategy in the decoding phase significantly reduces model parameters and computational load, and channel attention is used to obtain weighted features, ensuring model accuracy. Experimental results demonstrate that the lightweight segmentation approach proposed in this chapter exhibits significant improvements in accuracy and robustness compared to other methods.

## AUTHOR CONTRIBUTIONS

*Methodology*: Xiaoyan Wang, Jianhao Yu, Bangze Zhang; *Formal analysis and investigation*: Xiaoyan Wang, Bangze Zhang; *Writing—original draft preparation*: Jianhao Yu, Bangze Zhang; *Writing—review and editing*: Xiaoyan Wang; Xiaojie Huang, Ming Xia; *Funding acquisition*: Xiaoyan Wang, Xiaojie Huang, Ming Xia; *Resources*: Xiaojie Huang, Xiaoting Shen; *Supervision*: Xiaojie Huang, Ming Xia, Xiaoting Shen.

## CONFLICT OF INTEREST STATEMENT

The authors declare no conflicts of interest.

## Footnotes

1
https://competitions.codalab.org/competitions/17094

2
https://sds.fudan.edu.cn/ba/a0/c17412a178848/page.htm

3
https://challenge.isic‐archive.com/landing/2017/43/

4
https://datasets.simula.no/kvasir‐seg/
